# Using Remote, Automated Verbal Paired Associates for Learning Over Repeated Exposure: Six Learning Bursts Over Six Months

**DOI:** 10.1002/alz.092502

**Published:** 2025-01-03

**Authors:** Alexander J Kaula, Bronagh McCoy, Nick Taptiklis, Francesca K Cormack

**Affiliations:** ^1^ Cambridge Cognition, Cambridge United Kingdom; ^2^ University of Cambridge, Cambridge United Kingdom

## Abstract

**Background:**

Learning over repeated exposures (LORE) is an emerging paradigm that has shown sensitivity to AD biomarkers in apparently cognitively unimpaired (CU) samples using visual stimuli. We present results of a six‐month study in a CU sample that explored brief (<2 minute) assessment of verbal LORE alongside other measures of memory and sleep.

**Method:**

The Prolific platform was used to recruit 190 participants. In a baseline session we obtained a measure of sleepiness (Karolinska Sleepiness Scale), and visual and verbal measures of memory using paired associates learning (PAL) and verbal paired associates (VPA). Five‐day bursts of VPA‐LORE were then carried out once‐monthly for six months, with participants learning a new set of word‐pairs each burst. One month after the final burst, delayed recall for all 48 associates was tested in a single session.

The first session of a burst comprised presentation of the month’s associates, followed by a test of immediate recall. In subsequent sessions, cued‐recall was tested first, after which the set was presented again. A measure of LORE was created by integrating recall scores over the burst. We used linear mixed‐effects modelling to explore relationships among 1) age and baseline memory measures, measures of immediate recall and LORE and 2) between day‐by‐day scores, age and sleepiness.

**Result:**

Test‐retest correlations of LORE scores from burst‐to‐burst over the six bursts were strong (*rp* = .69 ‐ .75). At baseline, sleepiness did not affect PAL‐errors or VPA‐errors. PAL‐errors were positively associated with age (*β* = 0.391, *p*<.001) and VPA‐errors, but VPA‐errors were not associated with age. LORE scores were not significantly associated with age or PAL‐errors. However, day‐to‐day learning within a burst was impacted by daily sleepiness (*β* = ‐0.122, *p*<.001). In contrast to LORE, delayed‐recall after the final burst was significantly impacted by age (*β =* ‐0.010, *p* = .043).

**Conclusion:**

Reliability of VPA‐LORE was found to be good between bursts over six months. The association of LORE with PAL but not with age suggests VPA‐LORE measures a stable form of memory different from PAL. Additionally, the sensitivity the task to sleepiness may be relevant for use in AD‐related research given the impact of AD pathology on sleep.

